# Effects of motion direction and power of Er,Cr:YSGG laser on pull-out bond strength of fiber post to root dentin in endodontically-treated single-canal premolar teeth

**DOI:** 10.1186/s40824-019-0165-y

**Published:** 2019-11-15

**Authors:** Loghman Rezaei-Soufi, Leili Tapak, Mahsa Forouzande, Reza Fekrazad

**Affiliations:** 1 Department of Restorative Dentistry, Dental Research Center, Dental Faculty, Hamadan University of Medical Sciences, Hamadan, Iran; 2 Department of Biostatistics, School of Health, Hamadan University of Medical Sciences, Hamadan, Iran; 3 Department of Restorative Dentistry, Dental Faculty, Hamadan University of Medical Sciences, Hamadan, Iran; 4 Department of Periodontology, Laser Research Center in Medical Sciences, Dental Faculty, AJA University of Medical Sciences, Tehran, Iran

**Keywords:** Laser, Fiber post, Surface treatment

## Abstract

**Background:**

Inadequate retention and gradual debonding of intracanal post from root dentin is a major cause of failure of endodontically treated teeth restored with fiber post.

**Main body:**

This study aimed to assess the effect of surface treatment of quartz fiber posts with different powers and motion directions of Er,Cr:YSGG laser on their pull-out bond strength to root dentin in endodontically treated premolar teeth. In this study, 105 fiber posts were divided into 7 groups according to their surface treatment with different powers of Er,Cr:YSGG laser at 2780 nm wavelength, 20 Hz frequency and 150 μs pulse duration in circumferential (C) or longitudinal (L) motion directions: Control group (no treatment), 0.5 W laser in longitudinally (L0.5), 1.0 W laser in longitudinally (L1), 1.5 W laser in longitudinally (L1.5), 0.5 W laser in circumferentially (C0.5), 1.0 W laser in circumferentially (C1) and 1.5 W laser in circumferentially (C1.5). After cementation, pull-out bond strength was measured in Newton (N). Each sample was inspected under a stereomicroscope at × 25 magnification to determine the mode of failure. Two samples of each group were inspected under a scanning electron microscope (SEM). Data were analyzed using two-way ANOVA and Tukey’s test with significant level of 0.05. The pull-out bond strength of 0.5 W groups had significant differences with the control group (*P* = 0.009). The bond strength of 1.0 W and 1.5 W groups were not significantly different (*P* = 0.630) but were higher than the control and 0.5 W groups (*P* < 0.001). Motion direction of laser irradiation had no significant effect on the bond strength (*P* = 0.384). The interaction effect of power and motion direction of laser irradiation had no significant effect on the bond strength (*P* = 0.092).

**Conclusion:**

Fiber posts treated with 0.5, 1.0 and 1.5 W Er,Cr:YSGG laser showed higher bond strength to dentin compared to posts with no surface treatment. However, the motion directions of laser irradiation had no significant effect on the bond strength. In order to minimize damage to post surface and achieving maximum bond strength, longitudinal surface treatment of posts with 1.0 W power of Er,Cr:YSGG laser is recommended.

## Background

Endodontically treated teeth have often lost a large portion of their structure and require further reinforcement to enhance the strength and retention of restoration. To increase the retention of restoration, metal or fiber posts are used in the root canal of endodontically treated teeth. Fiber posts ease coronal restoration of endodontically treated teeth compared to cast posts and do not require laboratory fabrication [1]. Fiber posts have a modulus of elasticity similar to that of dentin and therefore, show optimal biomechanical behavior. Thus, treatment failure is mainly due to cement loss and reduction of retention [2].

Surface treatments are performed in order to overcome the problem of inadequate retention of fiber posts. Surface treatment of fiber posts with hydrogen peroxide significantly increases their shear bond strength [3, 4].

Sandblasting with 50 μm alumina particles increases the surface roughness of posts without compromising their integrity. Moreover, the post surface can be silanized to enhance its bond strength [5]. Also, evidence shows that heat-treated silane significantly increases the push-out bond strength of fiber posts to root dentin [6]. However, use of silane-sandblasting with aluminum oxide and plasma (NH_3_ and HMDSO) yields superior results compared to the application of 24% hydrogen peroxide [7].

Lasers have many applications in dentistry, for example in pulp diagnosis, dentinal hypersensivity, pulp capping and pulpotomy, sterilization of root canals, root canal shaping and obturation, apicectomy [8]. Laser irradiation improves the properties of materials such as better hardenability in non-precious alloys [9].

On the other hand, laser irradiation has been suggested as a fast modality for surface treatment of organic and inorganic materials as well as surface modification of biomaterials. Laser irradiation reportedly increases the bond strength of dental materials to tooth dentin. Laser irradiation creates a rough and irregular surface. Porosities on the surface of materials allow penetration of adhesive and creation of resin tags, which enhance micromechanical interlocking and retention between the post and adhesive [10].

Surface treatment of root dentin and fiber post and a combination of both may increase the retention of fiber post to root dentin. Use of Er:YAG laser in dental root canal treatment facilitates disinfection, shaping, cleansing, and roughening (affecting bond strength of the root canal) of the canal. Laser irradiation on the root surface increases the bonding strength of surface-treated prefabricated glass-fiber posts [11].

Evidence shows that root dentin treated with Er:YAG laser with 2940 nm and 1.5 W shows higher push-put bond strength to fiber post [12]. Nagase et al. evaluated the efficacy of root canal treatment with different lasers and stated that root canal treatment with Nd:YAG (neodymium-doped yttrium aluminum garnet laser) and Er,Cr:YSGG (erbium, chromium:yttrium-gallium-garnet laser) can effectively increase the bond strength of root dentin to intracanal posts [13]. However, Kirmali et al. showed that root dentin treatment with Er,Cr:YSGG laser of 1, 2 and 3 W does not increase the push-out bond strength of fiber post to root dentin [14].

Post Surface treatments with hydro fluoric acid, silica coating and Er:YAG laser (erbium-doped yttrium aluminium garnet laser) irradiation were found to be effective methods for improving the bonding of glass fiber posts to resin cement [15].

Comparison of air abrasion and 1.5 W power Er:YAG laser for fiber post surface treatment revealed the highest bond strength in posts treated with air abrasion [16]. However, further studies are needed to investigate the efficacy of laser surface treatment with different irradiation parameters [16]. Another study showed that treatment of fiber post with 2780 nm Er,Cr:YSGG laser (1.5 W) increased its push-out bond strength to root dentin in the entire canal length [17].

Kurt et al. showed that Er:YAG laser treatments on the FRC post surface decreased the bond strength. Airborne-particle abrasion and HF acid etching are alternative methods for increasing bond strength of FRC posts to composite core material [18].

Considering the current studies on the retention of fiber posts, study the effects of surface treatment of fiber posts with laser on their bond strength to root dentin seem to be imperative. This study aimed to assess the effect of surface treatment of quartz fiber posts with Er,Cr:YSGG laser with different powers and motion direction on their pull-out bond strength to root dentin in single-rooted, single-canal endodontically treated premolars. The null hypothesis stated that different powers and motion directions of Er,Cr:YSGG laser had no significant effect on the pull-out bond strength of fiber posts to root dentin.

## Materials and methods

### Tooth and post preparation

This in vitro experimental study, approved by research ethics committee of Hamadan University of medical science (IR.UMSHA.REC.1397.335), evaluated 175 single-rooted, single-canal mandibular premolars extracted for orthodontic reasons. After disinfection of the teeth and removal of soft tissue debris and calculus, the teeth were immersed in 0.1% thymol solution at 4 °C. The mesiodistal width of the teeth was measured at the cementoenamel junction using a digital caliper (CD-8”CSX; Mitutoyo). Of all, 105 teeth with equal dimensions were selected for this study [17]. Standardized peri apical radiographs were obtained from the teeth to assess the canal morphology. The teeth had straight roots and closed apices and had no cracks, caries, resorption, calcification or previous endodontic treatment. They had a minimum root length of 13 mm. All endodontic treatments were performed by the same experienced operator. Access cavity was first prepared and the pulp tissue was extirpated using a barbed broach (Dentsply Sirona). The actual root length was then directly measured using a file (#10 K-file; Dentsply Sirona). The file was introduced into the canal until its file tip was visible at the apex; 1 mm was subtracted from the actual length to determine the working length. The canals were instrumented using the step-back technique, and the apical region was prepared to master apical file #40. The coronal part of each canal was shaped with size 2 to 4 Gates-Glidden drills (A0008; Dentsply Sirona). After each instrumentation, the canals were rinsed with 2 mL of 5% sodium hypochlorite (EMPLURA®; Merck). The smear layer was removed using 5 mL of 18% ethylenediaminetetraacetic acid (EDTA; Ultradent) for 1 min. Finally, the canals were rinsed and dried with absorbent paper points (Dentsply Sirona). The canals were then filled with gutta-percha (Dentsply Sirona) and resin sealer (AH-Plus; Dentsply Sirona) using lateral condensation technique. The coronal part of the access cavity was temporarily restored with resin modified glass ionomer temporary restorative material (Fuji II LC; GC). The samples were then stored in distilled water at 4 °C for 2 days. Next, the tooth crowns were cut at the cementoenamel junction by a double-blade diamond disc using a high-speed hand-piece under water coolant. Next, 9 mm of gutta-percha was removed by #3 Gates-Glidden drill, and the post space was prepared using #2 DT drills (DT Light post; RTD Inc) with 9 mm length measured from the cementoenamel junction at the buccal surface of the root.

The roots and 105 #2 quartz fiber posts (DT Light post; RTD Inc) with 1.8 mm diameter at the coronal and 1 mm diameter at the apical region were randomly divided into 7 groups including one control and six experimental groups (*n* = 15).

In control group, no surface treatment was performed and in other groups the apical 9 mm of each post was irradiated with Er,Cr:YSGG laser (Waterlase iPlus; Biolase) with 2780 nm wavelength, 20 Hz frequency and 150 μs pulse duration via Z6 tip of gold hand-piece with 600 μm diameter and 10% water and 15% air. The post surface had 2 mm distance from the tip and was perpendicular to it. Laser irradiation was performed for 30 s. Power and motion direction of laser hand-piece tip was different between groups (Table 1). The groups subjected to longitudinal motion direction of laser irradiation were designated with “L” which means that the laser tip was moved longitudinally along the longitudinal axis of the post and fibers. Laser irradiation was repeated for the adjacent region until the entire post circumference was treated. The groups subjected to circumferential motion direction of laser irradiation were designated with “C” which means the motion direction of laser hand-piece tip was circumferential and perpendicular to the longitudinal axis of the post. The adjacent region was then irradiated until the entire post circumference was treated.

**Table 1:** Power and motion direction of groups

groups	control	L 0.5	C 0.5	L 1	C 1	L 1.5	C 1.5
power	–	0.5 W	0.5 W	1.0 W	1.0 W	1.5 W	1.5 W
direction	–	longitudinal	circumferential	longitudinal	circumferential	longitudinal	circumferential

In order to assess the morphology of the post surfaces, in each group two extra posts were treated according to the manufacturer’s instructions for inspection under a scanning electron microscope (SEM1450 VP; LEO).

### Fiber post cementation

For cementation of the posts in the prepared canals, the root canals were first rinsed with 10 mL of sterile saline to remove debris and root filling remnants from the canals and they were dried with absorbent paper. Dentin was conditioned with 37% phosphoric acid (Ultra-Etch; Ultradent) for 15 s, rinsed for 15 s and dried with absorbent paper. Adhesive (Adper Single Bond 2; 3 M ESPE) was applied into the canal by a microbrush and excess adhesive was removed by a paper point. It was light-cured with a LED light-curing unit (Bluephase; Ivoclar Vivadent) with a light intensity of 1200 mW/cm^2^. The output of LED light-curing unit had been previously controlled by a power meter. A dual-cure resin cement (Rely X ARC; 3 M ESPE) was mixed according to the manufacturer’s instructions and applied into the root canal using a #40 Lentulo. Resin cement was also applied to the surface of the posts, which had been disinfected with alcohol, rinsed and dried. Next, the post was inserted into the canal with moderate constant finger pressure for 30 s. After 3 min of primary chemical polymerization, excess cement was removed. The tip of the light-curing unit was in direct contact with the coronal end of the post and curing was performed through the post for 40 s using a LED light-curing unit.

### Thermocycling and pull-out bond strength test

The samples were stored in distilled water at room temperature for 24 h. Then, the specimens were subjected to thermocycling for 3000 cycles between 5 and 55 °C in water baths using a dwell time of 20 s. Next, before mounting of the roots, the apical two-thirds of the external root surface was roughened by 010 diamond fissure bur (Flat End Cylinder 837; Tizkavan) to increase the retention of the roots in acrylic resin for pull-out test. The roots with cemented posts were mounted in a PVC cylinder with 2 cm diameter and 2 cm height using a surveyor (Ney; Dentsply) such that the longitudinal axis of the posts was perpendicular to the horizon. Next, the space around the roots in the cylinder was filled with self-cure acrylic resin (Unifast Trade; GC).

For the pull-out test, first a hole was created in the lower third of the PVC cylinder for attachment to the inferior compartment of the universal testing machine (Z250; Zwick). Next, the coronal part of the posts was placed in the mandrel attached to the upper compartment of the universal testing machine and the pull-out test was performed at a crosshead speed of 1 mm/min. The maximum load value for each sample was recorded in Newton (N).

### Failure mode and morphology assessment

Each sample was inspected under a stereomicroscope (SIX16; Olympus) at × 25 magnification to determine the failure mode. The failure mode was classified into three groups of adhesive (between the post and resin cement such that no cement was observed on the post or between resin cement and root dentin such that the post was completely covered with cement), cohesive (in dentin or post) and mixed (resin cement had covered the post surface).

To assess the post surface morphology by SEM, each post was mounted on a metallic stub and gold sputter-coated by a sputter coater (SC7620; Quorum Technologies Ltd). They were then inspected under × 100 and × 500 magnifications. The SEM images were prepared from one-third middle of each post surface. This was done to assess the effect of laser irradiation on the morphology of the post surface and its fibers.

### Statistical analysis

Data were analyzed using SPSS version 24. The pull-out bond strength data of different groups were compared with two-way ANOVA and post-hoc Tukey’s test at 0.05 level of significance.

## Results

Table 2A shows the pull-out bond strengths of the 7 groups in Newton (N). According to the Kolmogorov-Smirnov test, the bond strength data had a normal distribution in the study groups (*P* > 0.05).

**Table 2:** Summary statistics of bond strength (Newton) for each group (A), two way ANOVA results (B) and post hoc analysis (C)

A) Statistics of bond strength				
Group	Mean	Std. Deviation	Minimum	Maximum
C1.5	241.5733 N	12.99546	219.70 N	262.10 N
L1.5	242.3867 N	12.46686	220.70 N	262.10 N
C1.0	240.9467 N	12.86978	220.20 N	262.70 N
L1.0	238.5933 N	10.97318	221.10 N	260.10 N
C0.5	222.9133 N	6.41826	209.90 N	230.70 N
L0.5	221.2067 N	7.34064	209.70 N	232.70 N
Control	218.3267 N	4.98691	209.60 N	229.40 N
Total	232.2781 N	14.15982	209.60 N	262.70 N
B) ANOVA table				
Source	F statistics	Df1	Df2	*P* value
Power	12.038	2	99	p < 0.001
Direction	0.764	1	99	0.384
Interaction	2.903	1	99	0.092
C) Post hoc analysis for Power				
Group (I)	Group (J)	Mean Difference (I-J)	Standard Error	*P* value
Power 0.5 W	Power 1.0 W	−11.990	2.836	*p* < 0.001
	Power 1.5 W	−13.794	3.563	*p*< 0.001
	Control	9.453	3.563	0.009
Power 1.0 W	Power 1.5 W	−1.803	3.737	0.630
	Control	21.443	3.737	*p* < 0.001
Power 1.5 W	Control	23.247	4.315	*p* < 0.001

**Fig. 1:**
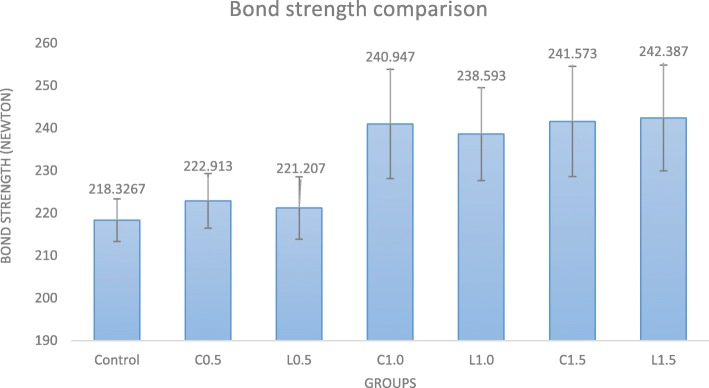
Comparison of bond strength

According to the results of ANOVA test reported in Table 2B, laser power had a significant effect on the pull-out bond strength (*P* < 0.001) but direction of laser irradiation had no significant effect on the bond strength (*P* = 0.384). The interaction effect of power and motion direction on bond strength was not significant (*P* = 0.092). Pairwise comparison of groups is presented in Table 2C. The pull-out bond strength of laser groups with 0.5 W power had significant differences with the control group (*P* = 0.009). The bond strength, in groups with 1.0 W and 1.5 W power, were not significantly different (*P* = 0.630) but in these groups the bond strength were significantly higher than those in the control group and 0.5 W power groups (*P* < 0.001). Motion direction (L or C) had no significant effect on the bond strength (*P* = 0.384). The interaction effect of laser power and motion direction had no significant effect on the bond strength either (*P* = 0.092) (see Fig. 1).

Table 3 shows the modes of failure in different surface treatment groups. Mixed failure had a higher frequency in all groups. In general, 14, 1.9 and 83% of all fractures were adhesive, cohesive and mixed, respectively.

**Table 3:** Comparison of different failure modes in different methods of laser surface treatment

Failure	Groups							Total
	C 1.5	L 1.5	C 1.0	L 1.0	C 0.5	L 0.5	Control	
Adhesive	2(13%)	1(7.6%)	2(13%)	2(13%)	3(20%)	3(20%)	2(13%)	15(14%)
Cohesive	0	0	0	0	0	1(7.6%)	1(7.6%)	2(1.9%)
Mixed	13(86%)	14(93%)	13(86%)	13(86%)	12(80%)	11(73%)	12(80%)	88(83%)
Total	15	15	15	15	15	15	15	105

**Fig. 2:**
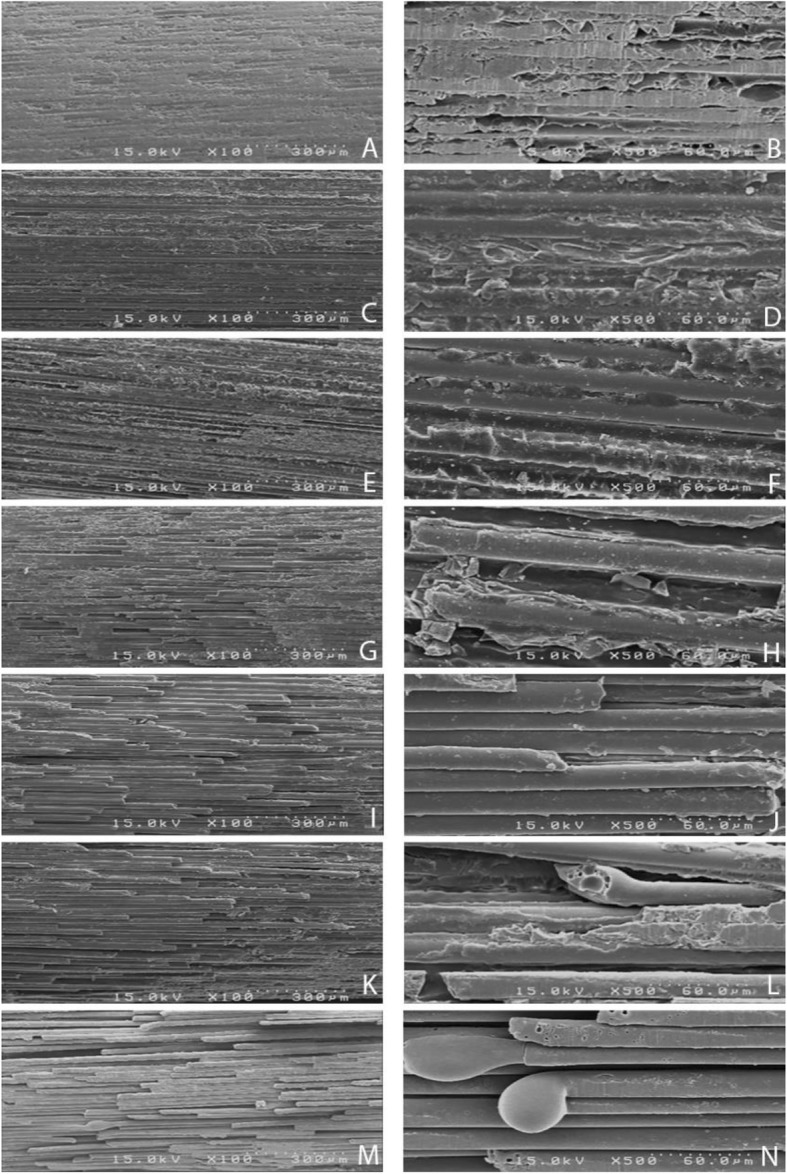
SEM micrographs of experimental groups with × 100 and × 500 magnifications. A, Control × 100. B, Control × 500. C, L0.5 × 100. D, L0.5 × 500. E, C0.5 × 100. F, C.05 × 500. G, L1 × 100. H, L1 × 500. I, C1 × 100. J, C1 × 500. K, L1.5 × 100. L, L1.5 × 500. M, C1.5 × 100. N C1.5 × 500

SEM of the samples at 100 and 500 magnifications revealed that higher powers of laser caused greater degradation of the superficial epoxy resin layer and higher fracture and destruction of fibers in fiber posts. In samples subjected to circumferential laser irradiation, deeper fibers were exposed and underwent melting and fracture (Fig. 2).

## Discussion

Fiber posts in endodontically treated teeth that have lost a great portion of their coronal structure significantly decreases the risk of their composite restorations failure [19]. They should have a strong bond to root dentin in order to be able to increase the retention of restorations because evidence shows that the bond strength of fiber posts to root dentin decreases after a period of function in the oral cavity [20].

In this study, the effects of power and motion direction of Er,Cr:YSGG laser used for surface treatment of fiber posts on the bond strength were evaluated. The results showed that fiber posts irradiated with Er,Cr:YSGG laser with 0.5, 1.0 and 1.5 W power yielded a higher bond strength to dentin compared to fiber posts with no surface treatment. Increasing the laser power (control group, 0.5 W and 1.5 W) further increased the bond strength but no significant difference was noted in bond strength values of 1.0 W and 1.5 W laser groups. However, the motion direction had no significant effect on the bond strength. Thus, the null hypothesis regarding no effect of power on the bond strength of fiber post to root dentin was rejected but the null hypothesis regarding no significant effect of motion direction on bond strength was accepted.

At this time, to assess the bond strength of fiber posts to root dentin, several methods are available. This study assessed the pull-out bond strength because it measures the total debonding value of post from the entire canal length and allows simultaneous assessment of shear and tensile stresses [21–23].

Self-adhesive resin cements show acceptable results for bonding of fiber posts to root dentin [24, 25]. Since resin cements have etch and rinse and self-etch bonding mechanisms, they provide a more favorable bond strength for cementation of fiber posts [26]. Thus, a resin cement in etch and rinse mode was used for bonding of fiber posts to root dentin.

Surface treatment of root dentin and fiber post and their combination can be used in order to increase the bond strength of fiber posts to root dentin.

The efficacy of root dentin treatment with different lasers has been previously evaluated [11–14]. Chemical and micromechanical treatment of the fiber posts surface or their combination may enhance their bonding properties. Several studies have aimed to find the best method for surface treatment of fiber posts.

In a study on the surface treatment methods, these procedures were categorized into three categories as 1) treatments that affect on the chemical bonding between a composite and post (coating with priming solutions); 2) treatments that intend to roughen the surface (sand-blasting and etching); 3) combine micromechanical and chemical components either by using the two above mentioned methods or a unique system (such as Co-Jet) [27].

In more recent studies, the role of lasers in surface treatment has been highlighted carefully. Erbium family lasers with high absorption are more commonly used for conditioning and creation of micro-scale porosities to enhance the bond strength.

Achammada et al. reported that laser treatment with Er:YAG and Er,Cr:YSGG, significantly improved the push-out bond strength of FRC posts luted with resin cement. They suggested that further studies should be carried out with different types and parameters of laser devices to study its effect on the push-out bond strength [28].

Arslan reported that irradiation of Er:YAG laser with 450 mJ energy and 10 Hz frequency for 60 s and 100 μs pulse duration increased the pull-out bond strength of fiber posts [29]. Gomes et al. reported that irradiation of Er,Cr:YSGG laser with 2780 nm wavelength, 150 mJ energy, 10 Hz frequency, 1.5 W power, 140 μs pulse duration and 60% water and 40% air for 60 s in non-contact mode increased the push-out bond strength of fiber posts [17]. Hashemikamangar et al. evaluated the effect of surface treatment of fiber posts with Er,Cr:YSGG laser and demonstrated that irradiation of Er,Cr:YSGG laser with 1.0 and 1.5 W power, 20 Hz frequency, 140 μs pulse duration in non-contact mode with 80% water and 60% air for 30 s enhanced the micro-push-out strength of fiber posts [30]. Kurtulmus-Yilm et al. reported that irradiation of Er,Cr:YSGG laser with 1.0 and 1.5 W power, 20 Hz frequency, 140 μs pulse duration and 80% water and 60% air for 30 s in non-contact mode increased the push-out bond strength of fiber posts [31].

Tuncdemir et al. reported that irradiation of Er:YAG laser with 2940 nm wavelength, 150 mJ energy, 10 Hz frequency,100 μs pulse duration for 60 s in non-contact mode without air or water cooling did not increase the bond strength [32]. Ghavami et al. concluded that irradiation of Er,Cr:YSGG laser with 20 Hz frequency, 140 μs pulse duration and 80% water and 60% air for 10 s in non-contact mode did not increase the bond strength of fiber post and even power of 1.5 W decreased the bond strength of post. Irradiation of 1.0 W power of laser had no significant difference with the control group [33]. Variation in matrix combination, types and structure of fiber posts, applied resin cements as well as bond strength test may be the reason of these inconsistent results.

SEM micrographs showed that higher powers of laser caused greater surface roughness, further eliminated the epoxy resin, further exposed the fibers in fiber posts and increased the risk of degradation and melting of post. But, 1.0 W laser improved the bond strength while caused less destruction of the post surface. In this study, laser in longitudinal and circumferential motions was used for surface treatment of fiber posts. This was done aiming to assess whether changing the motion direction of laser tip results in better retention. The results showed that in the laser with same power, changing the motion direction of laser irradiation had no significant effect on the bond strength. SEM micrographs revealed that fiber posts subjected to circumferential irradiation of laser which showed greater destruction of matrix and further exposure of deep fibers. Since both modes of irradiation acted similarly, longitudinal mode of irradiation is preferred.

Assessment of the modes of failure of different groups revealed that the mode of failure was mixed in most groups, and adhesive and cohesive modes of failure had a lower frequency, which indicates the presence of a strong bond between the cement and post. Ghavami-Lahiji et al. reported that the majority of failures were mixed [33]. However, in the study by Gomes et al. the majority of failures were adhesive between resin cement and fiber post or between resin cement and root dentin [17].

Despite the current findings and use of different powers and motion directions of laser irradiation, difficulty in maintaining the precise distance between the samples and laser hand-piece and speed of laser irradiation and difficulty in controlling the load applied to the samples along their longitudinal axis in pull-out test were among the limitations of this study. Here, we evaluated only one type of fiber posts. However, considering the variability in fiber posts available in the market in terms of type, density and orientation and length of fibers, it is suggested to assess the effect of laser on different types of fiber posts in future studies. Also, different laser types with variable physical parameters should be compared with Er,Cr:YSGG laser for surface treatment of fiber posts. Last but not least, surface treatment of fiber posts and root dentin with a combination of different methods should be tested in future studies to minimize surface destruction and obtain ideal results. To overcome the laboratory limitations, especially in thermocycling, it is favorable to use animal for future studies [34].

## Conclusion

As conclusion, fiber posts treated with 0.5, 1.0 and 1.5 W Er,Cr:YSGG laser at 2780 nm wavelength, 20 Hz frequency, 150 μs pulse duration, 10% water and 15% air yielded higher bond strength to root dentin compared to fiber posts with no any surface treatment. Increasing the laser power (control group, 0.5 W and 1.0 W) increased the bond strength but the bond strength 1.0 W and 1.5 W groups were not significantly different. Motion direction of laser irradiation had no significant effect on the bond strength. In order to minimize damage to the post surface and achieve maximum bond strength, 1.0 W laser in longitudinal mode is recommended for surface treatment of fiber posts.

## Data Availability

The datasets used and/or analyzed during the current study are available from the corresponding author on reasonable request.
